# Electronic Health Risk Behavior Screening With Integrated Feedback Among Adolescents in Primary Care: Randomized Controlled Trial

**DOI:** 10.2196/24135

**Published:** 2021-03-12

**Authors:** Laura Richardson, Elizabeth Oshrin Parker, Chuan Zhou, Julie Kientz, Elizabeth Ozer, Carolyn McCarty

**Affiliations:** 1 Seattle Children's Research Institute Seattle, WA United States; 2 Department of Pediatrics University of Washington Seattle, WA United States; 3 Department of Human Centered Design & Engineering University of Washington Seattle, WA United States; 4 Division of Adolescent and Young Adult Medicine Department of Pediatrics University of California, San Francisco San Francisco, CA United States; 5 Office of Diversity & Outreach University of California, San Francisco San Francisco, CA United States

**Keywords:** adolescent health services, primary care

## Abstract

**Background:**

Health risk behaviors are the most common sources of morbidity among adolescents. Adolescent health guidelines (Guidelines for Preventive Services by the AMA and Bright Futures by the Maternal Child Health Bureau) recommend screening and counseling, but the implementation is inconsistent.

**Objective:**

This study aims to test the efficacy of electronic risk behavior screening with integrated patient-facing feedback on the delivery of adolescent-reported clinician counseling and risk behaviors over time.

**Methods:**

This was a randomized controlled trial comparing an electronic tool to usual care in five pediatric clinics in the Pacific Northwest. A total of 300 participants aged 13-18 years who attended a well-care visit between September 30, 2016, and January 12, 2018, were included. Adolescents were randomized after consent by employing a 1:1 balanced age, sex, and clinic stratified schema with 150 adolescents in the intervention group and 150 in the control group. Intervention adolescents received electronic screening with integrated feedback, and the clinicians received a summary report of the results. Control adolescents received usual care. Outcomes, assessed via online survey methods, included adolescent-reported receipt of counseling during the visit (measured a day after the visit) and health risk behavior change (measured at 3 and 6 months after the visit).

**Results:**

Of the original 300 participants, 94% (n=282), 94.3% (n=283), and 94.6% (n=284) completed follow-up surveys at 1 day, 3 months, and 6 months, respectively, with similar levels of attrition across study arms. The mean risk behavior score at baseline was 2.86 (SD 2.33) for intervention adolescents and 3.10 (SD 2.52) for control adolescents (score potential range 0-21). After adjusting for age, gender, and random effect of the clinic, intervention adolescents were 36% more likely to report having received counseling for endorsed risk behaviors than control adolescents (adjusted rate ratio 1.36, 95% CI 1.04 to 1.78) 1 day after the well-care visit. Both the intervention and control groups reported decreased risk behaviors at the 3- and 6-month follow-up assessments, with no significant group differences in risk behavior scores at either time point (3-month group difference: β=−.15, 95% CI −0.57 to −0.01, *P*=.05; 6-month group difference: β=−.12, 95% CI −0.29 to 0.52, *P*=.57).

**Conclusions:**

Although electronic health screening with integrated feedback improves the delivery of counseling by clinicians, the impact on risk behaviors is modest and, in this study, not significantly different from usual care. More research is needed to identify effective strategies to reduce risk in the context of well-care.

**Trial Registration:**

ClinicalTrials.gov NCT02882919; https://clinicaltrials.gov/ct2/show/NCT02882919

## Introduction

### Background

Health risk behaviors, such as alcohol use, risky sexual behaviors, and low physical activity, are among the most common causes of morbidity and mortality during adolescence and young adulthood [1,2]. To reduce risk and morbidity, adolescent preventive care guidelines recommend screening and counseling to reduce these behaviors as a component of annual well-care visits [3,4]*.* However, the delivery of preventive screening is inconsistent, and only a small proportion of screened adolescents report having received counseling to reduce risk with rates of counseling varying by type of behavior [2,5-7].

Research has shown that the use of standardized screening methods, including electronic screening tools, can increase screening delivery, detection of risk, and adolescent-reported clinician counseling [6,8,9]*.* Adolescents report greater comfort in disclosing behaviors with electronic screening methods compared with other methods [10-13]. However, few studies have examined the impact of increasing clinician counseling on adolescent behavior outcomes in the context of multi-risk screening, as is commonly performed in well-care visits. In a recent review article examining multi-risk screening in adolescents, 9 studies were identified, with some demonstrating effects on risk behaviors [7]. Among these trials, variations in intervention duration, intensity, behaviors studied, and impacted outcomes led to a limited ability to draw definitive conclusions. In addition, based on the studies in this review, the magnitude of behavioral changes was small to modest, and the only risk behavior for which change was found in more than one study was for an increase in bicycle helmet use.

When administered consistently, electronic screening can serve to reduce biases related to selecting who gets screened and how questions are asked [14]. In addition, some studies suggest that adolescents are more likely to use preventive health services when they are given information electronically about health behaviors [15-18]. Furthermore, 2 studies found that the use of electronic screening improved adolescents’ perceptions of clinician communication and partnership [19,20].

### Study Aim

**Figure 1:**
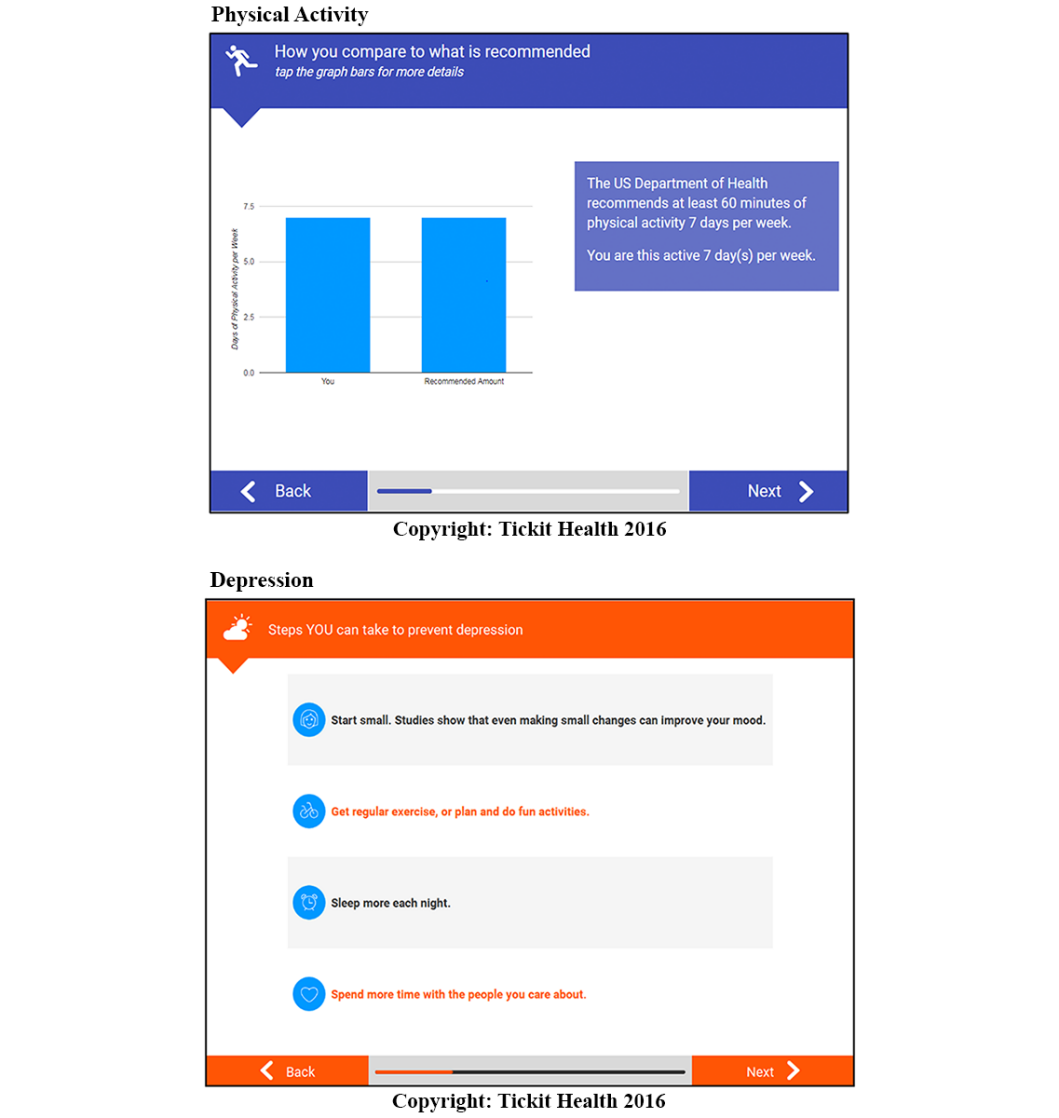
Screenshots of the personalized feedback from the Check Yourself tool by behavior.

In this study, we aim to examine the efficacy of a tool delivered via an app or website link that combined electronic screening with integrated personalized motivational feedback. The interactive tool was developed with adolescents’ input and designed to be administered before a well-care visit to prepare adolescents to discuss risk behavior change with their clinicians when indicated. The tool tested in this study (Figure 1; see additional examples in Multimedia Appendix 1) is a modified version of a previously tested tool [21]. The modifications were made to increase youth engagement with the tool and increase the ease with which clinicians can interpret the results with changes based on adolescent and clinician input gathered through a human-centered design process [22]. The tool also generated a printed one-page clinician summary of adolescent-reported behaviors. The primary outcomes of interest were adolescent-reported clinician counseling during the visit, health risk behaviors at 3 months, and patient satisfaction. The secondary outcome was health risk behavior at 6 months. We hypothesized that the intervention would increase clinician counseling and reduce health risk behaviors at 3 months.

## Methods

### Overview

We conducted a parallel-group randomized controlled intervention study comparing the electronic screening and feedback tool to usual care in the context of a well-care visit. Adolescent participants (aged 13-18 years) were recruited from 5 pediatric clinics in Washington State between September 30, 2016, and January 12, 2018. Clinics were contacted via the Puget Sound Pediatric Research Network and invited to participate based on interest in the study and the number of adolescent patients served. Clinics were located in urban, suburban, and small city locations. The providers of these clinics included physicians and advanced practitioners. Residents and other trainees were not included in the study because of concerns about continuity in the clinical setting. Among participating clinics, the average monthly number of adolescents aged 13 to 18 years with scheduled well-visits was 49 (SD 17; range 31-71).

Sites were added to the study on a rolling basis with the goal of recruiting a minimum of 60 adolescents per site for a total recruitment goal of 300 adolescents. The study sample size was predetermined by the study statistician with the goal of having 80% power to detect an effect size as small as 0.3. Enrollment goals were met and exceeded at 4 of the 5 participating clinics, resulting in a total sample of 301 adolescents. The fifth clinic began enrollment late and, after entering the study, determined that they were not comfortable sharing patient contact data for research team outreach in the manner approved by the institutional review board. As a result, only a small number of patients were invited to participate, of whom 2 were enrolled.

Study outreach procedures included clinics sharing contact information for all adolescent patients between the ages of 13 and 17 years who were scheduled for an upcoming well-care visit. The study staff coordinated the mailing of a letter to parents from the clinic, inviting eligible adolescents to participate in the study and providing a phone number to opt out of further contact. This letter was followed by a phone call from the study staff to provide further information and assess eligibility. Exclusion criteria included planning to cancel the well-care visit, being out of the study age range, having a sibling who was previously invited to participate, lacking phone or internet access, or if the adolescent did not speak English.

Parental consent and adolescent assent were obtained via phone for participants aged 13-17 years, whereas participants aged 18 years provided direct consent. The consent forms stated that the study would compare electronic screening with feedback and provision of the results to the clinician to electronic screening alone (with no feedback or results provided to the clinician). Although most of the adolescents approached for the study spoke English, some parents did not. To support the inclusion of these adolescents, recruitment and parental consent materials were translated into Spanish and Somali, the most common languages spoken in participating clinics.

Before beginning recruitment at each clinic, the study statistician developed a computer-generated list of random numbers that was entered into REDCap [23] with a 1:1 allocation schema stratified by age (13-15 or 16-18 years), male or female gender (as provided by the study clinics based on their records), and clinic. Participant randomization occurred after the completion of consent and assent procedures and before data collection*.* Adolescents were not told their study assignment but could potentially determine it based on whether or not they received feedback as part of the baseline assessment. After consent was completed, adolescent participants were sent an online link for their baseline screening assessment, with or without integrated feedback, based on the study assignment. Baseline data were collected online with phone support by trained study staff before the well-care visit. As part of the procedures, control and intervention adolescents were instructed to complete their respective baseline screening components in a private setting where they could respond confidentially. All procedures for recruitment were approved by the Seattle Children’s Institutional Review Board before starting study activities. The study protocol is available upon request from the corresponding author.

### Intervention Procedures

Intervention adolescents completed electronic screening with integrated personalized feedback, and their clinician received a printed one-page summary report of the screening results. The electronic screening tool assessed protective factors and risk behaviors using a HEADSS pneumonic (Home, Education, Activities, Drugs, Depression, Sexuality, and Safety) framework [24]. The tool was also screened for specific nutritional behaviors (sweetened beverage intake and fruit and vegetable intake), physical activity, and sleep. The integrated feedback component was designed to deliver messages that increased motivation and self-efficacy for healthy behavior. Feedback content varied according to behavior assessed and the youth-reported risk level. It included a combination of education, tips for change, and motivational messaging, including positive reinforcement for adolescents who did not engage in risks and messages to motivate behavior change when risks were present using a combination of normative feedback comparing adolescent-reported risks to peer reports, guidelines, and goal setting.

The tool in this study is an adapted version of the Check Yourself tool (version 2) [21,25], revised to increase interactive features with input from adolescent users, clinicians in collaboration with faculty, and researchers in human-centered design. Specific changes that were developed with adolescent input include increasing image-based feedback versus text, adding functionality to allow participants to choose to see more versus less information on each topic, and the option to receive more information about topics of interest in the form of a one-time text or email. In addition, screening content was modified to add response options related to gender identity, remove screen time assessments, enhance screening for depression and anxiety, and enhance screening and new feedback related to marijuana use. On the basis of internal tracking data, the tool took an average of 15 (SD 8) minutes to complete among control adolescents and 18 (SD 10) minutes among intervention adolescents who also received integrated feedback.

The one-page paper clinician summary included a dashboard with flags categorizing the adolescent health risks as low, moderate, or high within 6 areas: nutrition, activity, substance use, emotions, sexual activity, and safety. Individual screening responses were provided below the dashboard for each area so that clinicians could examine which specific behaviors resulted in a flag. Risk behavior severity categories (high, moderate, and low) were defined a priori based on health guidelines or expert consensus (Multimedia Appendix 2) and integrated into the electronic screening algorithms. The study staff coordinated with each clinic to develop protocols so that clinicians would receive the summary report before the visit.

### Control Procedures

Control adolescents completed the electronic screening portion of the tool as a baseline assessment but did not receive integrated feedback. The clinicians did not receive any screening results.

From the outset of the study, clinicians were instructed to continue their standard health risk screening procedures for all patients (intervention and control). The standard processes for all 5 sites included a combination of paper intake screeners and interviews during the visit to assess risk, but the content of the paper screeners varied. One clinic used standardized paper anxiety and depression screens. Another clinic used a self-designed form that asked about sleep and safety risks, including texting while driving, driving under influence, helmet use, and seatbelt use. Outside of these 2 examples, there was no overlap between the health risk behavior content in the electronic screening tool and the standard screening forms employed by study clinics. None of the clinics employed a standard form to screen for confidential health risk behaviors, such as sexual activity or drug use. All of the clinics indicated that their providers asked about confidential risk behaviors during the well-care visit, although data were not available regarding the consistency of these practices.

Before enrollment, all clinicians received an invitation to complete a 15-minute online training module to orient them to the electronic tool and how to interpret the clinician summary. As randomization was at the patient level, clinicians could be exposed to both intervention and control patients.

### Surveys

The baseline assessment consisted of responses from the electronic screening tool (with or without feedback depending on assignment) conducted before the well-visit (details provided in Multimedia Appendix 3). In addition, all adolescents completed online follow-up surveys 1 day, 3 months, and 6 months following their well-care visit. The 1-day follow-up survey assessed the content of the visit, including the delivery of counseling to change behavior for each screened behavior. Items assessing the visit were adapted from the Adolescent Report of the Visit developed by Ozer et al [26]. The 3-month and 6-month follow-up surveys assessed the same health risk behaviors as at baseline, collected via an online survey tool, REDCap [23]. Participants were asked about suicidality at baseline and at all follow-up time points. To ensure safety, study investigators, who are also clinicians, followed up with all participants in either study arm who reported having thoughts of harming themselves in the past 2 weeks and thoughts of killing themselves or suicide attempts in the past 3 months and assisted them in accessing clinical services.

### Analysis

All data analyses were conducted using *R 3.5.0* [27] using an intent-to-treat framework. We first conducted bivariate analyses to evaluate differences in demographics and baseline risk between adolescents in the control and intervention group. Subsequently, we conducted our main analyses on the 3 primary outcome measures: clinician counseling during the visit, a summary score of health risk behaviors measured at 3 months after the visit, and patient satisfaction. Our secondary outcome measure, the health risk behaviors summary score at 6 months was analyzed together with the 3-month summary score using repeated measures analysis.

On the basis of the study design, missing data only occurred during the outcome assessments. We compared the baseline characteristics of participants with and without missing outcomes and found no differences between the groups. We further conducted sensitivity analyses for each of our primary outcomes using multiple imputation with chained equations (MICE) methods using linear regression and predictive mean matching for continuous outcomes. For categorical outcomes, we applied classification and regression tree methods for imputation using MICE methods. Estimates from the fitted models on multiple imputed data sets were pooled to generate the final results for inference. In conducting these sensitivity analyses, we found that the results were almost identical for the imputed and complete case analysis. Thus, only the complete case analysis results are presented in this paper.

### Clinician Counseling Outcome

Clinician counseling during the visit, measured on the 1-day assessment, was defined as adolescent report of the clinician having counseled them to change an endorsed behavior toward health. This measure was constructed by summing all endorsed moderate- and high-risk behaviors for which adolescents reported receiving counseling. We conducted an adjusted analysis using a mixed effects Poisson regression model in which the dependent variable was the counseling measure, and the treatment group was the predictor of interest. Baseline age and sex were included as covariates, and a clinic-specific random effect was included to account for clustering within the clinic. The total number of endorsed moderate- and high-risk behaviors was entered as an offset to ensure that the regression coefficients had proper rate interpretation. As an exploratory subanalysis to evaluate whether higher-risk behaviors were more likely to receive counseling than moderate-risk behaviors, we also conducted 2 additional regression analyses focused specifically on counseling for each category of risk behaviors: high-risk and moderate-risk behaviors, controlling for the same variables as the main analysis.

### Risk Behavior Outcome

The risk behavior outcome analyses employed a summary score of all assessed behaviors at 3 months (primary outcome) and 6 months (secondary outcome) after the visit. The risk behavior scores were constructed for each participant by adding all of the risk behaviors for which the tool included feedback (alcohol use, marijuana or other drug use, driving while intoxicated, tobacco use, depression, texting while driving, inconsistent seatbelt use, inconsistent helmet use, unprotected sexual activity, high sugary beverage intake, low fruit and vegetable intake, inadequate sleep, and low physical activity) at 3 and 6 months. High-risk behaviors were assigned a score of 2, moderate-risk behaviors were assigned a score of 1, and low-risk behaviors were assigned a score of 0 (score potential range: 0-21, further details regarding items in Multimedia Appendix 2). Treating baseline, 3-month, and 6-month risk scores as repeated measures, we applied linear mixed effects regression models to compare changes over time in adolescent-reported total risk scores at 3 and 6 months, relative to baseline, in intervention versus control adolescents controlling for baseline sex, age*,* and clinic as a random effect*.* To examine the effects of the intervention on health risk behaviors, we conducted exploratory logistic regression analyses for individual risk behaviors. Owing to concerns about estimate instability, we did not conduct analyses for individual behaviors in which fewer than 10 adolescents per study arm endorsed the behavior.

### Patient Satisfaction Outcome

Patient satisfaction was measured on the 1-day postvisit survey using a satisfaction scale ranging from 1 to 10 from the Consumer Assessment of Health care Providers and Systems [28].

Differences between groups were examined using linear mixed effect regression while controlling for baseline age, sex, and clinic-specific random effects.

Control adolescents were the reference group for all regression analyses. For mixed effects Poisson regression, we determined that an estimate was statistically significant if its 95% CI for the rate ratio did not include 1. For the mixed effects linear regression models, statistical significance was based on *P* values calculated using the Satterwaite degrees of freedom method [29].

## Results

### Overview

In total, letters were sent to 1665 homes inviting adolescents to participate (Multimedia Appendix 4). The final study sample that completed all consent and baseline procedures was 301 adolescents (301/1586, 18.9% of the eligible sample). One adolescent withdrew from the study and requested that their data not be used, leaving an analytic sample of 300 adolescents. After consent, 145 patients were randomized to the intervention group and 155 to the control group. The response rates at 1 day, 3 months, and 6 months were 94% (282/300), 94.3% (283/300), and 94.6% (284/300), respectively.

### Baseline Demographics and Risk Assessment

Randomization was balanced, with no differences between intervention and control adolescents in terms of demographics or baseline risk score (Table 1). Among the 300 participants, 43% (n=129) were female, 76% (n=228) were between the ages of 13 and 15 years, and 24% (n=72) were aged 16-18 years. Most adolescents identified as White (n=192, 64%), with the next largest group identifying as being of more than one race or “other” (n=55, 18.3%). In total, 92% (n=276) of adolescents had at least one health risk behavior at baseline, with a mean baseline risk score of 2.86 (SD 2.33) for intervention and 3.10 (SD 2.52) for control participants.

Table 2 summarizes the reported risk behaviors in order of baseline frequency at baseline, 3 months, and 6 months, with the most common risk behavior being low fruit and vegetable intake and the least frequent being driving under influence.

**Table 1:** Sample demographics.

Characteristic		Control (n=155)	Intervention (n=145)
**Gender, n (%)**			
	Female	70 (45.2)	59 (40.7)
	Male	82 (52.9)	86 (59.3)
	Trans or nonbinary	3 (1.9)	0 (0.0)
**Age (years), n (%)**			
	13-15	114 (73.5)	114 (78.6)
	16-18	41 (26.4)	31 (21.3)
**Race or ethnicity, n (%)**			
	White	99 (63.9)	93 (64.1)
	Hispanic	12 (7.7)	7 (4.8)
	African American	13 (8.4)	6 (4.2)
	Asian or Pacific Islander	7 (4.5)	7 (4.8)
	Native American	0 (0.0)	1 (0.7)
	Other or more than one	24 (15.5)	31 (21.4)
Risk behavior score at baseline, mean (SD)		3.10 (2.52)	2.86 (2.33)

**Table 2:** Prevalence of individual risk behaviors over time in intervention and control adolescents.

Behavior	Intervention			Control			Logistic regression, *P* value^a^
	Baseline (n=145), n (%)	3 months (n=138), n (%)	6 months (n=139), n (%)	Baseline (n=155), n (%)	3 months (n=145), n (%)	6 months (n=145), n (%)	
Low fruit or vegetable intake	115 (79.3)	106 (76.8)	98 (70.5)	132 (85.1)	118 (81.4)	111 (76.6)	.93
Low sleep time	46 (31.7)	52 (37.7)	65 (46.8)	54 (34.8)	55 (37.9)	72 (49.6)	.89
Low physical activity	39 (26.9)	44 (31.9)	36 (25.9)	50 (32.3)	52 (35.9)	51 (35.2)	.77
Inconsistent helmet use	37 (25.5)	24 (17.4)	22 (15.8)	39 (25.2)	25 (17.2)	20 (13.8)	.54
High sugary beverage intake	28 (19.3)	39 (28.2)	36 (25.9)	36 (23.2)	37 (25.5)	35 (24.1)	.47
Depression	13 (9.0)	15 (10.9)	14 (10.1)	23 (14.8)	15 (10.3)	18 (12.4)	.24
Inconsistent seatbelt use	16 (11.0)	7 (5.1)	11 (7.9)	10 (6.5)	7 (4.8)	10 (6.9)	.11
Texting while driving	9 (5.8)	10 (7.2)	8 (5.8)	13 (8.4)	8 (5.5)	10 (6.9)	^—b^
Marijuana use	10 (6.9)	4 (2.9)	3 (2.2)	7 (4.5)	4 (2.8)	5 (3.4)	—
Alcohol use	8 (5.5)	3 (2.2)	4 (2.9)	6 (3.9)	4 (2.8)	4 (2.8)	—
Tobacco use	4 (2.8)	0 (0.0)	1 (0.7)	4 (2.6)	2 (1.4)	3 (2.1)	—
Sexual risk	1 (0.7)	3 (2.2)	3 (2.2)	4 (2.6)	2 (1.4)	3 (2.1)	—
Driving under the influence	0 (0.0)	0 (0.0)	0 (0.0)	2 (1.3)	1 (0.7)	0 (0.0)	—

^a^*P* values were based on the likelihood ratio test comparing mixed effects logistic regression with and without period-by-group interaction. Both models controlled for random effects corresponding to within-individual clustering.

^b^Owing to concerns about estimate instability, we did not conduct analyses for individual behaviors in which fewer than 10 adolescents per study arm endorsed the behavior.

### Clinician Counseling Analysis Results

Among control adolescents, 380 moderate- and high-risk behaviors were endorsed, among which adolescents reported receiving clinician counseling for 148 (38.9%) behaviors during the visit. Intervention adolescents reported a total of 326 moderate- and high-risk behaviors, among which 184 (56.4%) were counseled by clinicians during the visits. In the Poisson regression analyses, intervention adolescents were significantly more likely to report that they had received counseling for their endorsed moderate- and high-risk behaviors than control adolescents (adjusted rate ratio [aRR] 1.36, 95% CI 1.04 to 1.78). To examine the impact of the intervention on rates of counseling by risk behavior severity level, we also examined rates of counseling for intervention and control adolescents based on whether they were classified as low, moderate, or high risk. Intervention adolescents were 40% more likely than adolescents in the control group to have received counseling for moderate-risk behaviors (aRR 1.40, 95% CI 1.09 to 1.80). For high-risk behaviors, the rate of counseling was 70% higher among intervention than control adolescents (aRR 1.70, 95% CI 1.06 to 2.74). There were no significant differences between intervention and control adolescents in reported counseling for no-/low-risk behaviors (aRR 1.12, 95% CI 0.85 to 1.48).

### Risk Behavior and Patient Satisfaction Analyses

**Figure 2:**
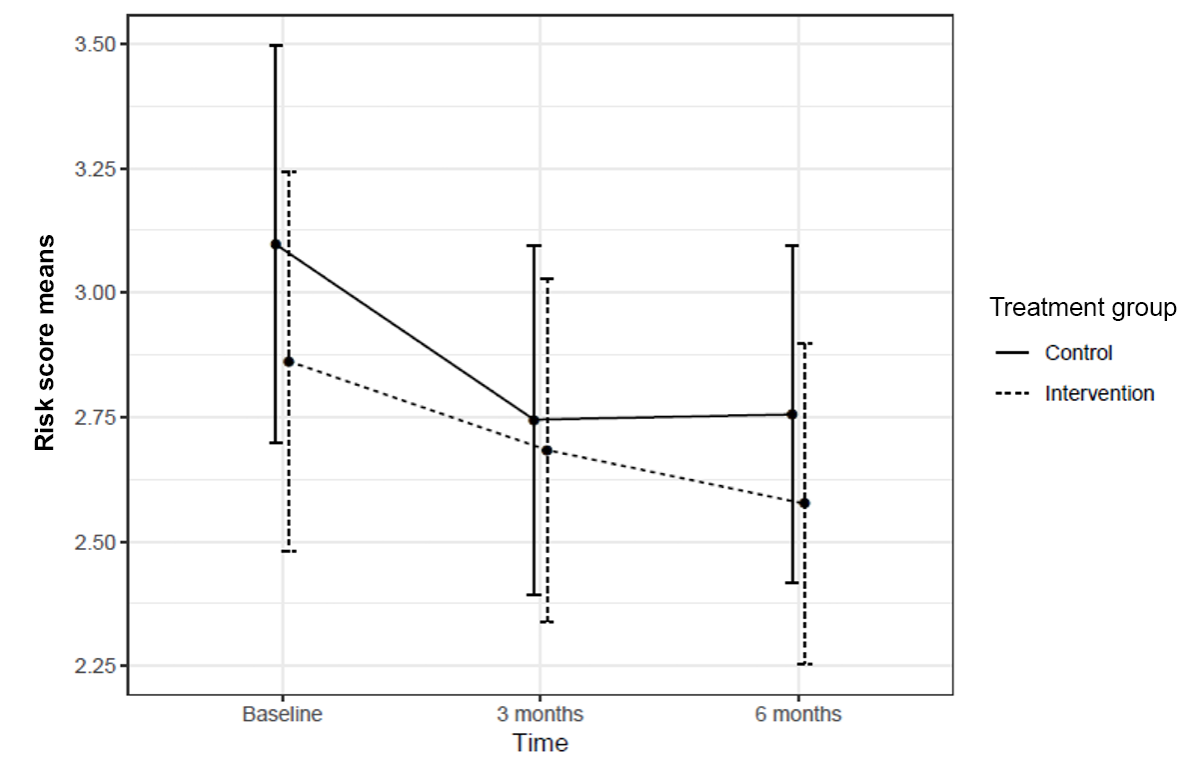
Health risk behavior scores in adolescents in the intervention and control groups by time.

The baseline risk score was 2.86 (SD 2.33) for adolescents in the intervention group and 3.10 (SD 2.52) for adolescents in the control group, respectively (*P*=.40). At 3 months, the risk score for adolescents in the intervention group was 2.68 (SD 2.04) compared with 2.74 (SD 2.11), respectively, for adolescents in the control group (*P*=.81). At 6 months, the risk score for adolescents in the intervention group was 2.58 (SD 1.87) compared with 2.76 (SD 2.05) for adolescents in the control group (*P*=.45). In mixed effects linear regression analysis including both 3- and 6-month outcomes, there was a significant reduction in risk behaviors in both groups at 3 months (β=−.33; 95% CI −0.62 to −0.05; *P*=.02) and 6 months (β=−.29; 95% CI −0.57 to −0.01; *P*=.05). There were no significant differences in risk scores between the intervention and control groups at either time point (Figure 2). At 3 months, the score difference between groups was 0.15 (β=−.15; 95% CI −0.25 to 0.55; *P*=.47), and at 6 months, it was 0.12 (β=−.12; 95% CI −0.29 to 0.52; *P*=.57). In secondary analyses examining individual behaviors, no significant differences in the reduction of behaviors were observed between the adolescents of the intervention and control groups (Figure 2). There were also no significant differences between groups in patient satisfaction with the well-care visit process based on regression analysis controlling for age, gender, and clinic as a random effect (intervention mean: 9.46, SD 0.79; control mean 9.27, SD 0.86; *P*=.07).

## Discussion

### Principal Findings

In this study of an integrated screening and feedback tool, Check Yourself version 2, we found that adolescents in the intervention group were significantly more likely to report having been counseled by clinicians on risk behaviors than adolescents in the control group. However, despite significant differences in reported counseling between adolescents in the intervention and control groups, both groups demonstrated reductions in risk behavior scores, and there were no significant differences between the groups at 3 or 6 months after the intervention. There were also no significant differences in satisfaction between the 2 groups. These results are in contrast to our original study [21], which showed both an increase in reported counseling and a reduction in risk behavior scores at 3 months for youth in the intervention group as compared with controls. This study further adds to the growing body of literature on multi-behavior screening and preventive counseling interventions in adolescent well-care visits, which suggests that although provider counseling can be increased, the effects on risk behavior reductions are modest and inconsistent across studies [7].

In comparing the results to our prior study, it is important to note that this study tested a modified version of the tool with increased interactive content, which allowed adolescents to control the amount of information they received. Although adolescents requested incorporating these choices in content viewing, it is possible that the new adaptations resulted in less content exposure, particularly for at-risk adolescents who were not concerned about their behaviors. In this study design, we did not have the ability to assess how long adolescents spent on specific components of the feedback, although we do know that the overall time spent in this version of the tool was longer than the prior tool version. Future studies of interactive eHealth tools such as this could provide a better understanding of how risk influences engagement in feedback content.

It is also important to note that both the intervention and control groups experienced decreases in risk 3 months following their well-care visit in this sample. The reduction in risk in the control group would have weakened the ability to detect a difference. At baseline, all of the clinics in the study indicated that they conducted some form of paper and interview assessment of risk behaviors during the well-care visit. We collected information on the paper tools implemented and did not find substantial overlap with the risk behavior screening content of the electronic tools; however, all control teens completed an electronic health risk behavior assessment as part of the baseline study procedures. Although the results of this screening were not provided to the clinicians, it is possible that even in the absence of feedback, participating in the electronic screening may have resulted in behavior change, as teens reflected on their responses to risk behavior questions. In addition, as participants were randomized at the individual level, it is possible that some of the improvement in the control group was due to spillover effects as study clinicians and clinic staff applied learning from working with adolescents in the intervention group to control group. Our clinician counseling measure was based on adolescent self-report and did not allow us to directly assess the content of counseling delivered during the visits to test this possibility.

Unlike our prior study, we also collected 6-month outcomes that allowed us to examine the long-term effects of the intervention. Although it was encouraging to see that risk scores continued to trend downward for the intervention sample, the differences between the control and intervention groups were not significant. Given the lack of effect at 3 months, it is difficult to draw conclusions from the 6-month data. Furthermore, 2 prior studies that examined both short-term (3 months) and long-term outcomes (12 months) found that significant differences in risk behaviors noted at 3 months were no longer significant at 12 months [30,31]. These 2 studies employed different models of brief interventions. One involved 9 hours of clinician training in motivational interviewing and system support for the implementation of a screening tool for all visits among eligible adolescents and young adults [30]. The second intervention focused on those aged 14 and 15 years enrolled in 8 general practice sites who were invited to participate in a 20-minute health consultation on risk behaviors of their choosing with a trained nurse [31]. Other studies that have examined 6- or 12-month outcomes have found significant improvements in single outcomes—helmet use [32,33] and exercise [19] only. Given the health care resources directed at screening and preventive counseling, understanding the long-term impacts of multiple risk behavior interventions is an area worthy of future study.

### Limitations

This study had several limitations. First, although the use of a combined risk behavior outcome measure allowed us to test across the full range of behaviors for which clinicians were providing counseling, it is more difficult to interpret. We selected this measure as we feel it is more consistent with the multi-risk focus of behavioral counseling delivered in the pediatric well-care visit setting. However, this approach limits the conclusions we can draw regarding changes in any specific behavior. We conducted secondary analyses of individual behaviors to allow for a more ready interpretation of the intervention; however, for many behaviors, the prevalence at baseline was too low to draw conclusions on behavior change. The use of this multi-risk measure also limits our ability to compare outcomes with other studies, as prior research has measured a range of individual behavior outcomes [7].

A second limitation of this study is the low prevalence of individual behaviors. Consistent with other studies in pediatric primary care [34,35], including our own [21], adolescents receiving well-care tended to be younger: 76% (228/300) of participants were in the 13- to 15-year-old age group. Younger adolescents are less likely to engage in risk behaviors than older adolescents, which may limit their ability to show changes in behaviors. It is also possible that adolescents are less likely to endorse risk in the setting of a well-child visit because of concerns about confidentiality. Our research with this tool in a school-based clinic setting demonstrated significantly higher rates of youth-reported risk behaviors even after matching for age [36]. Prior research has also suggested that acute visits may be a more effective platform for risk screening among adolescents [37,38]. To increase the effective delivery of counseling, more research is needed to identify the best venues for reaching older and at-risk adolescents, including the added benefits and costs of screening at acute visits as well as screening in school-based health settings. Finally, this study was conducted among adolescents who visited primary care clinics in the Pacific Northwest and may not be generalizable to other settings.

### Conclusions

Despite these limitations, this study adds to the literature regarding the use of eHealth tools in screening and preventive care for adolescents and raises important questions worthy of further study. Health risk behaviors have a significant influence on morbidity and mortality during adolescence and adulthood and guidelines recommend screening and intervention during adolescent well-care visits. Electronic screening has been repeatedly shown to increase provider identification of risk. This study further demonstrates that the addition of feedback for adolescents and results for clinicians increases clinician counseling. Electronic platforms such as these can be important tools for future research to examine the impact of components and types of preventive content to effect behavior change as well as how to reach the adolescents who would most benefit.

## Appendix

Multimedia Appendix 1 Check Yourself (version 2) screenshots.

Multimedia Appendix 2 Risk behaviors included in overall summary outcome measure.

Multimedia Appendix 3 Check Yourself app overview.

Multimedia Appendix 4 CONSORT (Consolidated Standards of Reporting Trials) diagram.

## Abbreviations

aRR: adjusted rate ratio
MICE: multiple imputation with chained equations
