# Supplementary material for: Electronic Health Risk Behavior Screening With Integrated Feedback Among Adolescents in Primary Care: Randomized Controlled Trial
Source: J Med Internet Res. 2021 Mar 12;23(3):e24135. doi: 10.2196/24135 (PMC7998326; doi:10.2196/24135)
Supplement: Multimedia Appendix 1 [file jmir_v23i3e24135_app1.pdf]

Copyright: Tickit Health 2016

SCREENING FOR SAFETY

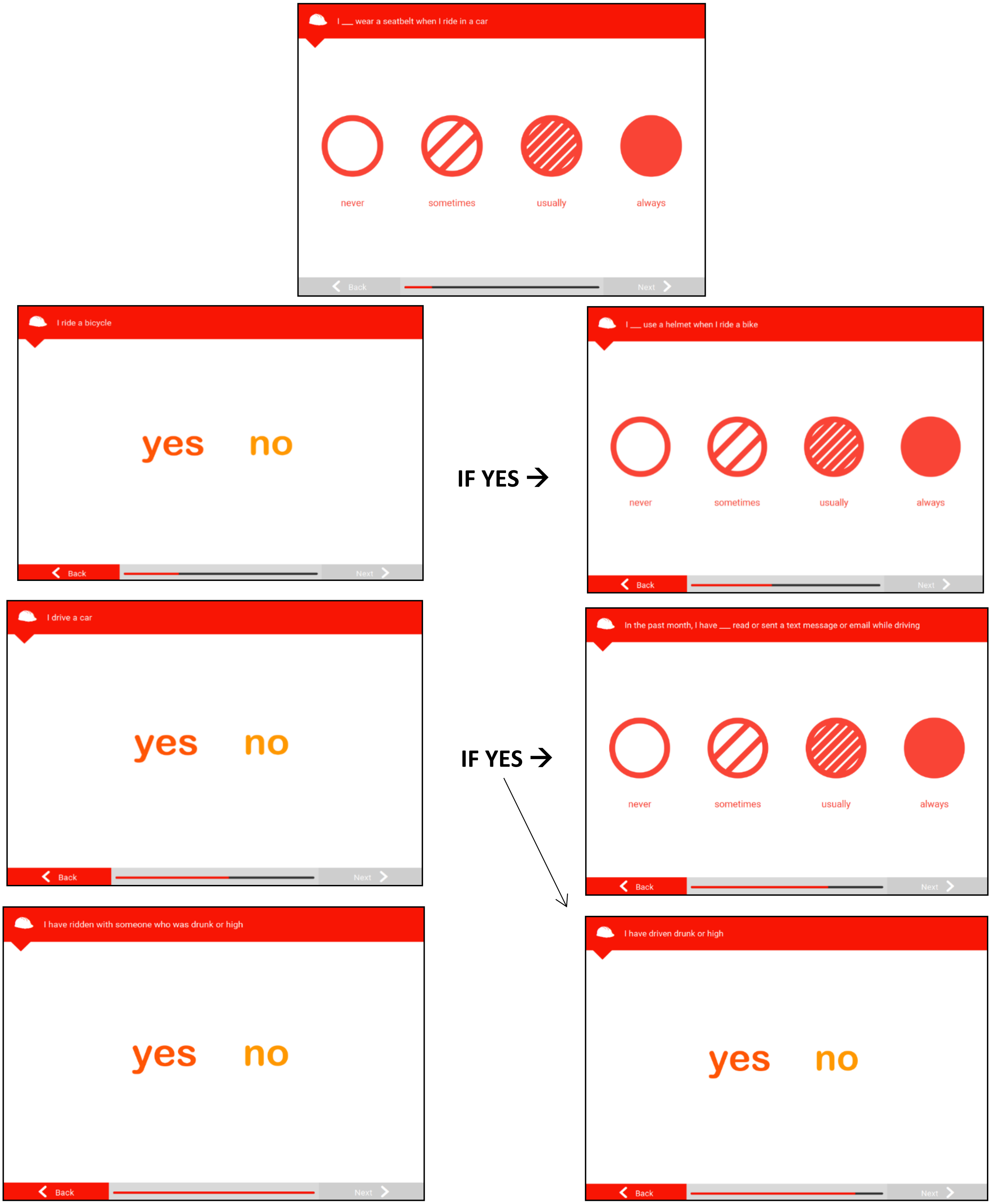

Copyright: Tickit Health 2016

FEEDBACK

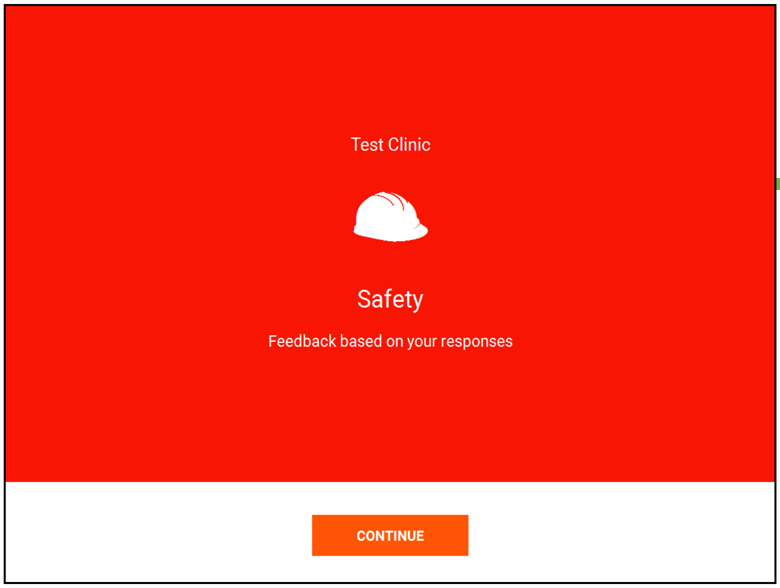

RISK

Helmet, Seatbelt, Driving

If user reports any other answer besides "never" AND

If user reports driving under the influence or riding with a drunk or high person

LOW RISK

Helmet, Seatbelt, Driving

If user reports seatbelt use and helmet use always AND

If user never reports texting while driving, driving under the influence, or riding with a drunk or high person

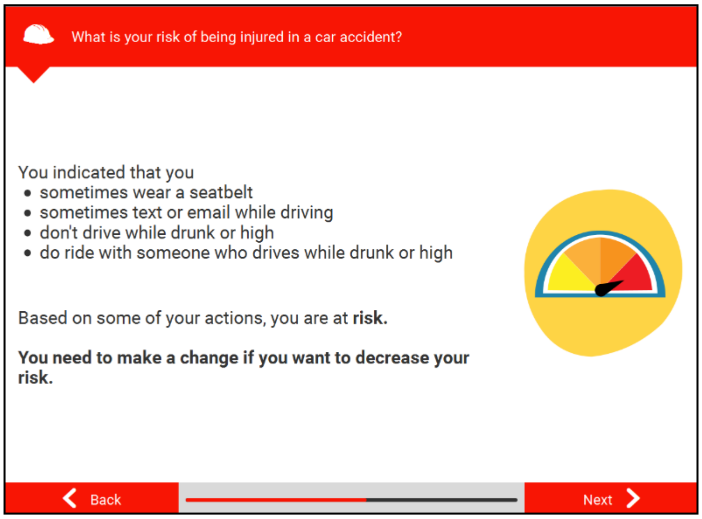

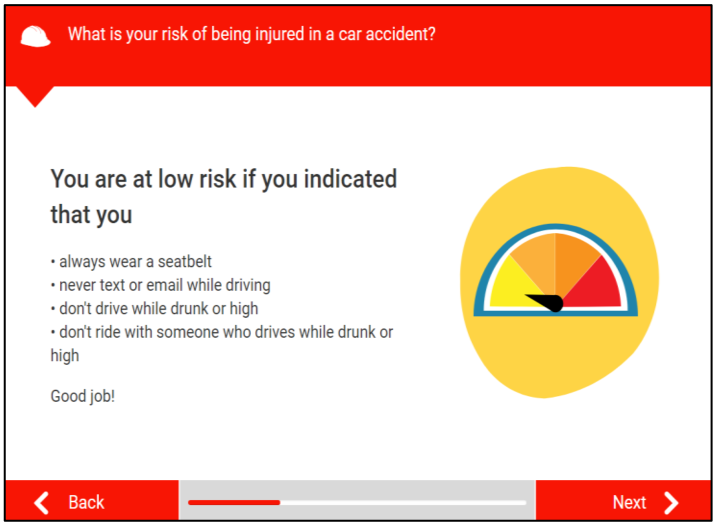

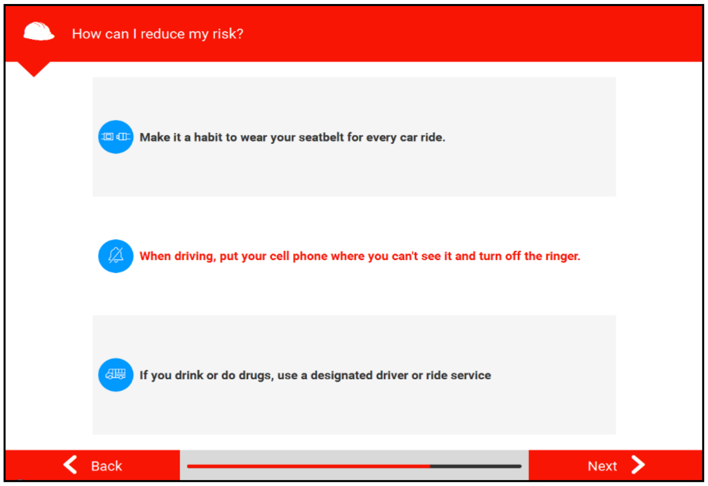

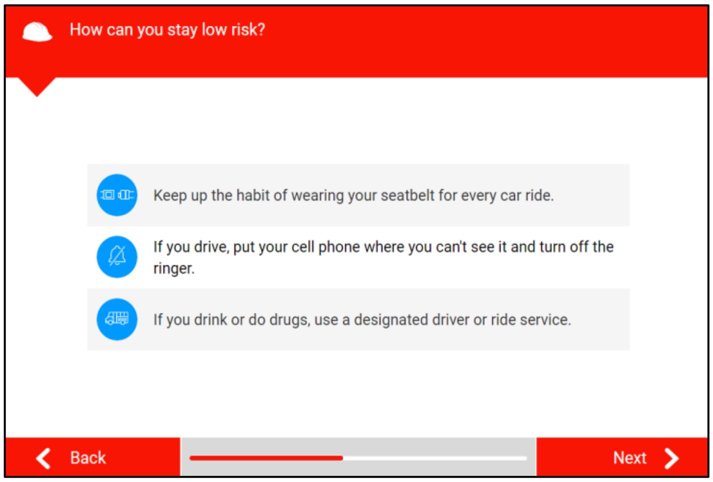

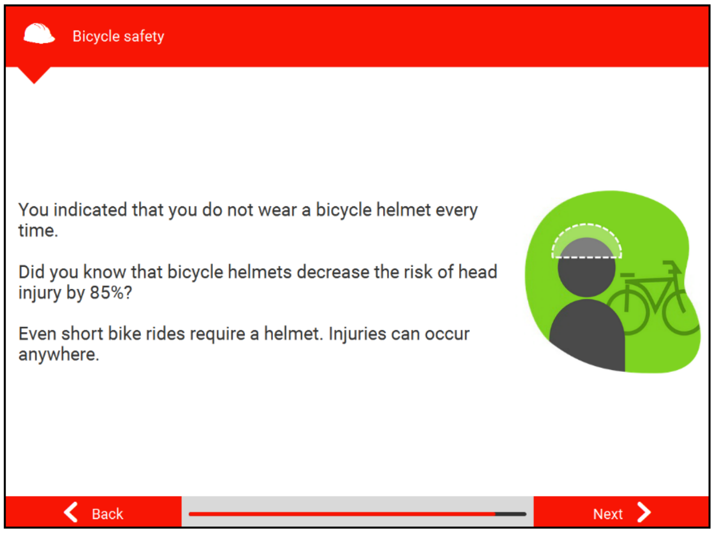

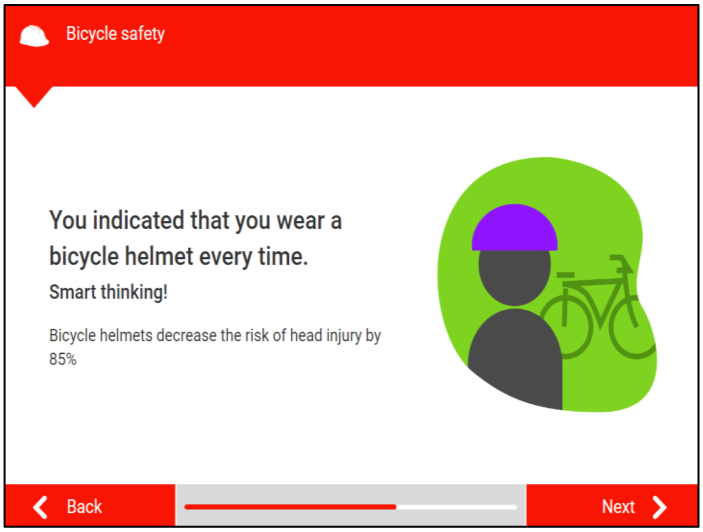

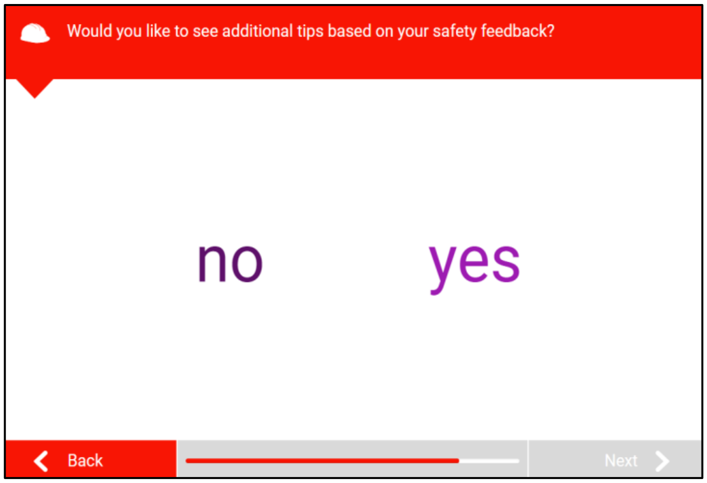

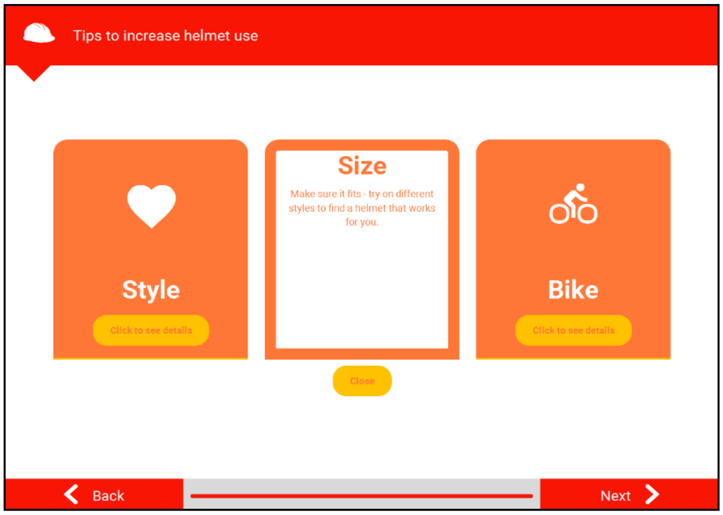

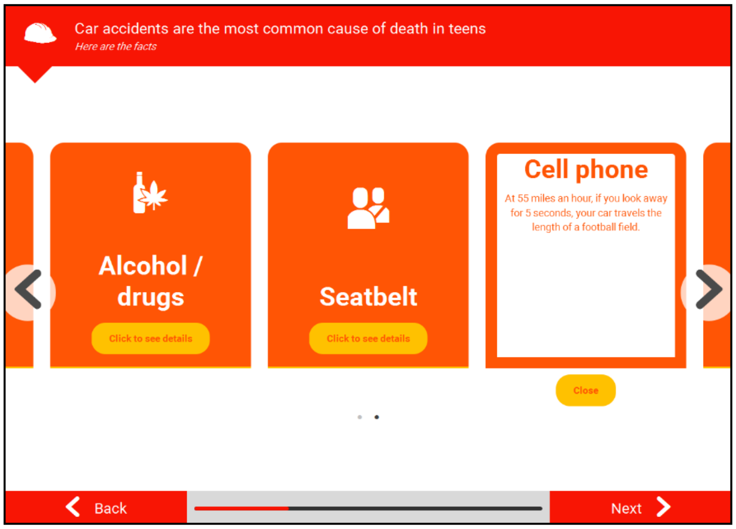

Copyright: Tickit Health 2016
