# Supplementary material for: Electronic Health Risk Behavior Screening With Integrated Feedback Among Adolescents in Primary Care: Randomized Controlled Trial
Source: J Med Internet Res. 2021 Mar 12;23(3):e24135. doi: 10.2196/24135 (PMC7998326; doi:10.2196/24135)
Supplement: Multimedia Appendix 2 [file jmir_v23i3e24135_app2.pdf]

Table 1 – Risk Behaviors Included in Overall Summary Outcome Measure

	Low (0)	Moderate (1)	High (2)
**Nutrition**			
Fruits/vegetables per day	4+	0-3	
Sugared drinks per day	0-1	2+	
**Activity**			
Sleep (typical hours/night)	8+	0-7	
Days/week physically active for at least 60 min	4+	0-3	
**Safety**			
Seat belt use	Always		Any other answer
Bike helmet use	Always		Any other answer
Drives drunk or high	No		Yes
Texts while driving	No		Yes
**Alcohol & Drugs**			
Alcohol#	Low risk for both alcohol frequency and quantity	Moderate risk on alcohol frequency and low risk on quantity	High risk on either alcohol frequency or quantity
Marijuana use (past 30 days) or Other drug Use	None	Moderate risk (1 day among 16-18 yo)	High risk: (Any use for 13-15 yo, ≥2 days for 16-18 yo) OR Use of any Other Drugs
Tobacco use	None	Any use	
**Sexual Health**			
Risky sexual behavior	Use birth control at last vaginal sex (any method) AND Always uses barrier method with sex		No birth control with last vaginal sex OR Does not always use barrier method with sex
**Depression**			
PHQ-9 score	<10		10+

**High-risk behaviors were defined a priori as those with a potential near-term risk of morbidity or mortality.

#Alcohol risk definitions were based on NIAAA guideline age-specific drinking frequency criteria and Donovan age- and gender-specific quantity of drinks per episode criteria. Specific criteria include: 13-15 year old high risk (no moderate risk for this age group): >0 days/month of drinking, >3 drinks/episode for males ages 14-15, or >2

drinks/episode for females/13 year old males. 16-17 year old high risk: >3 days/month of drinking, $\geq$5 drinks/episode for males or $\geq$3 drinks/episode for females. 16-17 year old moderate risk: >2 days/month, typical quantity less than high risk criteria. 18 year old high risk: >4 days/month of drinking, $\geq$5 drinks per episode for males, or $\geq$4 drinks/episode for females. 18 year old moderate risk: $\leq$ 3 days/month of drinking, typical quantity less than high risk criteria.
