# Supplementary material for: Electronic Health Risk Behavior Screening With Integrated Feedback Among Adolescents in Primary Care: Randomized Controlled Trial
Source: J Med Internet Res. 2021 Mar 12;23(3):e24135. doi: 10.2196/24135 (PMC7998326; doi:10.2196/24135)
Supplement: Multimedia Appendix 3 [file jmir_v23i3e24135_app3.pdf]

Check Yourself App Overview

The Check Yourself Screening App is designed to help primary care providers (PCPs) screen for important health risk behaviors prior to an adolescent well-child or sports physical visit. It covers seven broad areas that follow a HEADDSS assessment including: Home/Education, Activity, Healthy Eating, Sexual Health, Safety, Alcohol and Drug use, and Depression/Self-harm. Immediately following screening, adolescents are provided with personalized feedback, a motivational interviewing technique to support increasing motivation to prevent and reduce risk behaviors. Additionally, a 2-page provider report is generated for the PCP, with information about risk by area and individual items endorsed. Our color-coded risk snapshot follows these conventions:

- Green (check) indicates the patient meets the guidelines and has low/no risk of morbidity.
- Yellow (triangle) means the patient is not meeting the guidelines for healthy behavior.
- Red (X) means the patient is at high risk for morbidity due to behavior

Area	Screening	Color-Coded Risk	Adolescent Feedback
Home and education	10-15 questions:<br>Gender, age, race and family structure<br>Academic performance, missed days of school, relationships within the family and the presence of trusted adults and supports<br>Top two goals for the year	None	None
Activity	3 questions[1]:<br>Physical activity and sports<br>Amount of sleep	**Green:** Reasonable amounts of sleep and activity.<br><br>**Yellow:** Too little sleep or activity.	**All:** Tips and reasons for being or staying active and getting adequate sleep.<br><br>**Green:** Positive reinforcement of healthy activity.<br><br>**Yellow:** Information about the benefits and consequences of adequate sleep and activity.

[1] Prochaska JJ, Sallis JF, Long B. A physical activity screening measure for use with adolescents in primary care. *Arch Pediatr Adolesc Med.* May 2001;155(5):554-559.

Healthy Eating	4 questions:<br>Weight and body image[2])<br>Fruit and vegetable consumption[3]<br>Sugar sweetened beverage consumption[4]	**Green:** Eating the recommended amounts of fruits, vegetables, and sugary drinks, negative eating disorder (ED) screen.<br><br>**Yellow:** Dissatisfied with weight/body, drinks several sugary drinks or not eating enough fruits and vegetables.<br><br>**Red:** Positive report of unhealthy dieting practices or purging.	**All:** Information on what is a serving of fruits and vegetables and benefits of healthy eating. Alternatives to sugary drinks and nutritional information regarding sugar content of beverages.<br><br>**Green:** Positive reinforcement of healthy eating habits.
Sexual Health	2-6 questions derived from prior research work[5,6,7]:<br>Sexuality, sexual activity and sexual risk<br>Birth control and/or condom use	**Green:** Not having sex, or consistently using birth control and condoms.<br><br>**Red:** Not consistently using condoms, worried about pregnancy or STDs, not using birth control (females).	**All:** suggestion to discuss sexual health with their provider, information about STDs, best prevention methods for STDs and pregnancy, and effectiveness information about birth control options.<br><br>**Red:** Information about condoms and encouragement to use.

[2] American Medical Association. Guidelines for Adolescent Preventive Services Middle-Older Adolescent Questionnaire. Child and Adolescent Health Program, 1997. https://www.aap.org/en-us/advocacy-and-policy/aap-health-initiatives/Pages/Provider-Office-Tools.aspx. Accessed December 30, 2020.

[3] Prochaska JJ, Sallis JF. Reliability and validity of a fruit and vegetable screening measure for adolescents. *J Adolesc Health.* Mar 2004;34(3):163-165. DOI: 10.1016/j.jadohealth.2003.07.001.

[4] Healthy Youth Survey 2016 Analytic Report. Washington State Department of Social and Health Services, Department of Health, Office of the Superintendent of Public Instruction, and Liquor and Cannabis Board, June 2017.

[5] Ahrens KR, McCarty CA, Courtney ME, Dworsky A, Simoni J. Psychosocial pathways to sexually transmitted infection (STI) risk among youth transitioning out of foster care: Evidence from a longitudinal cohort study. *J Adolesc Health.* Oct 2013;53(4):478-85. DOI:10.1016/j.jadohealth.2013.05.010.

[6] Voisin DR, Hotton A, Tan K, Diclemente R. A Longitudinal Examination of Risk and Protective Factors Associated with Drug Use and Unsafe Sex among Young African American Females. *Child Youth Serv Rev.* Sep 1 2013;35(9):1440-1446.

[7] Martinez GM, & Abma, JC. (2015, July). Sexual Activity, Contraceptive Use, and Childbearing of Teenagers Aged 15-19 in the United States. NCHS Data Brief, July 2015, 209. http://www.cdc.gov/nchs/data/databriefs/db209.pdf. Accessed June 27, 2016.

Safety	3-7 questions[8]:<br>Seatbelt use<br>Helmet use<br>Texting while driving<br>Driving or riding with someone under the influence	**Green:** Screened negative for unsafe behaviors.<br><br>**Red:** Screened positive for unsafe behaviors.	**Green:** Positive reinforcement for healthy choices, tips for how to remain low risk.<br><br>**Red:** Facts about the frequency of car accidents among teens and tips to reduce risks while driving or riding a bike.
Alcohol and Drugs	4-20 questions[9,10,11]:<br>Tobacco use<br>Marijuana use<br>Other drug use<br>Normative perceptions<br>Reasons for use<br>Consequences of use<br>Readiness Ruler	**Green:** No substance use or low risk use (for older adolescents).<br><br>**Yellow:** Moderate risk drinking or use of tobacco or marijuana.<br><br>**Red:** High risk drinking, marijuana use, and/or other drug use.	**All:** Normative comparisons, facts about the potential harm of marijuana use and binge drinking.<br><br>**Green:** Positive reinforcement for not using.<br><br>**Yellow & Red:** Common risks of alcohol use, tips to reduce substance use, health effects of using, assess the effect of use on goals for the year.

[8] Centers for Disease Control and Prevention. National Youth Risk Behavior Survey. http://www.cdc.gov/HealthyYouth/yrbs/index.htm. Accessed October 31, 2013.

[9] Washington Stated Department of Health. The Healthy Youth Survey (HYS). 2010. http://www.doh.wa.gov/DataandStatisticalReports/HealthBehaviors/HealthyYouthSurvey.aspx. Accessed November 12, 2020.

[10] NIAAA Practitioners Guide http://pubs.niaaa.nih.gov/publications/Practitioner/YouthGuide/YouthGuide.pdf. Accessed November 19, 2020.

[11] Kuntsche E & Kuntsche S. Development and Validation of the Drinking Motive Questionnaire Revised Short Form (DMQ–R SF). *J Clin Child Adolesc Psychol.* 2009;38:6, 899-908, DOI: 10.1080/15374410903258967.

Depression and self-harm	16-18 questions:<br>Depression[12] (PHQ-9)<br>Anxiety[13] (GAD-7)<br>Suicidal Ideation screen items from the PHQ-A[14]	**Green:** No depression or suicidal ideation endorsed.<br><br>**Yellow:** Positive screen on GAD-7.<br><br>**Red:** Positive depression screen (PHQ-9), or suicidal ideation endorsed.	**Green:** Positive feedback, notified to talk to doctor if mood changes and tips to prevent depression.<br><br>**Yellow & Red:** Information about treatment options, identify activities to improve mood including identifying barriers and needed support.

[12] Richardson LP, McCauley E, Grossman DC, et al. Evaluation of the Patient Health Questionnaire-9 Item for detecting major depression among adolescents. *Pediatrics.* Dec 2010;126(6):1117-1123. DOI: 10.1542/peds.2010-0852.

[13] Kroenke K, Spitzer RL, Williams JBW, Monahan PO, and Löwe B. Anxiety disorders in primary care: prevalence, impairment, comorbidity, and detection. *Ann. Intern. Med*. 2007;146, 317–325. DOI: 10.7326/0003-4819-146-5-200703060-00004.

[14] PHQ - 9: Modified for Teens. American Academy of Child & Adolescent Psychiatry. Published 2010. https://www.aacap.org/App_Themes/AACAP/docs/member_resources/toolbox_for_clinical_practice_and_outcomes/symptoms/GLAD-PC_PHQ-9.pdf. Accessed December 16, 2020.
