# Supplementary material for: Electronic Health Risk Behavior Screening With Integrated Feedback Among Adolescents in Primary Care: Randomized Controlled Trial
Source: J Med Internet Res. 2021 Mar 12;23(3):e24135. doi: 10.2196/24135 (PMC7998326; doi:10.2196/24135)
Supplement: Multimedia Appendix 4 [file jmir_v23i3e24135_app4.pdf]

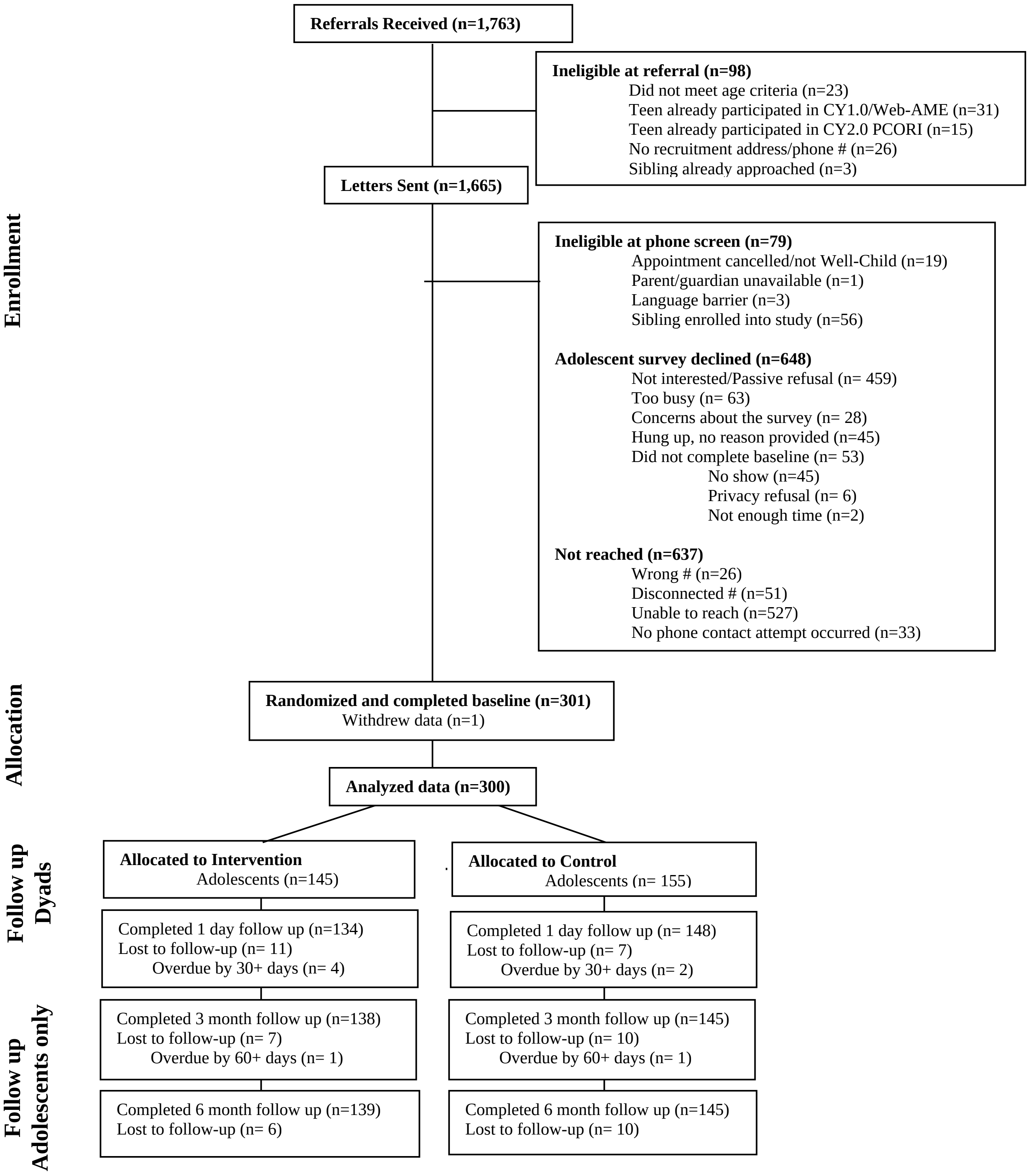
Referrals Received (n=1,763)
Ineligible at referral (n=98)
Did not meet age criteria (n=23)
Teen already participated in CY1.0/Web-AME (n=31)
Teen already participated in CY2.0 PCORI (n=15)
No recruitment address/phone # (n=26)
Sibling already approached (n=3)
Letters Sent (n=1,665)
Enrollment
Ineligible at phone screen (n=79)
Appointment cancelled/not Well-Child (n=19)
Parent/guardian unavailable (n=1)
Language barrier (n=3)
Sibling enrolled into study (n=56)
Adolescent survey declined (n=648)
Not interested/Passive refusal (n= 459)
Too busy (n= 63)
Concerns about the survey (n= 28)
Hung up, no reason provided (n=45)
Did not complete baseline (n= 53)
No show (n=45)
Privacy refusal (n= 6)
Not enough time (n=2)
Not reached (n=637)
Wrong # (n=26)
Disconnected # (n=51)
Unable to reach (n=527)
No phone contact attempt occurred (n=33)
Allocation
Randomized and completed baseline (n=301)
Withdrew data (n=1)
Analyzed data (n=300)
Follow up Dyads
Allocated to Intervention
Adolescents (n=145)
Allocated to Control
Adolescents (n= 155)
Completed 1 day follow up (n=134)
Lost to follow-up (n= 11)
Overdue by 30+ days (n= 4)
Completed 1 day follow up (n= 148)
Lost to follow-up (n= 7)
Overdue by 30+ days (n= 2)
Follow up Adolescents only
Completed 3 month follow up (n=138)
Lost to follow-up (n= 7)
Overdue by 60+ days (n= 1)
Completed 3 month follow up (n=145)
Lost to follow-up (n= 10)
Overdue by 60+ days (n= 1)
Completed 6 month follow up (n=139)
Lost to follow-up (n= 6)
Completed 6 month follow up (n=145)
Lost to follow-up (n= 10)
